# Metal Doping of Strongly Confined Halide Perovskite Nanocrystals under Ambient Conditions

**DOI:** 10.1021/jacs.5c03629

**Published:** 2025-05-05

**Authors:** Zachary A. VanOrman, Mateo Cárdenes Wuttig, Antti-Pekka M. Reponen, Taek-Seung Kim, Claire E. Casaday, Dongtao Cui, Tejas Deshpande, Huygen J. Jöbsis, Pascal Schouwink, Emad Oveisi, Aurélien Bornet, Christian Reece, Sascha Feldmann

**Affiliations:** † Rowland Institute, Harvard University, Cambridge, Massachusetts 02142, United States; ‡ Institute of Chemical Sciences and Engineering, École Polytechnique Fédérale de Lausanne, Lausanne 1015, Switzerland; § Department of Chemistry and Chemical Biology, Harvard University, Cambridge, Massachusetts 01238, United States; ¶ X-ray Diffraction and Surface Analytics Platform, École Polytechnique Fédérale de Lausanne, Sion 1950, Switzerland; # Interdisciplinary Centre for Electron Microscopy (CIME), École Polytechnique Fédérale de Lausanne, Lausanne 1015, Switzerland

## Abstract

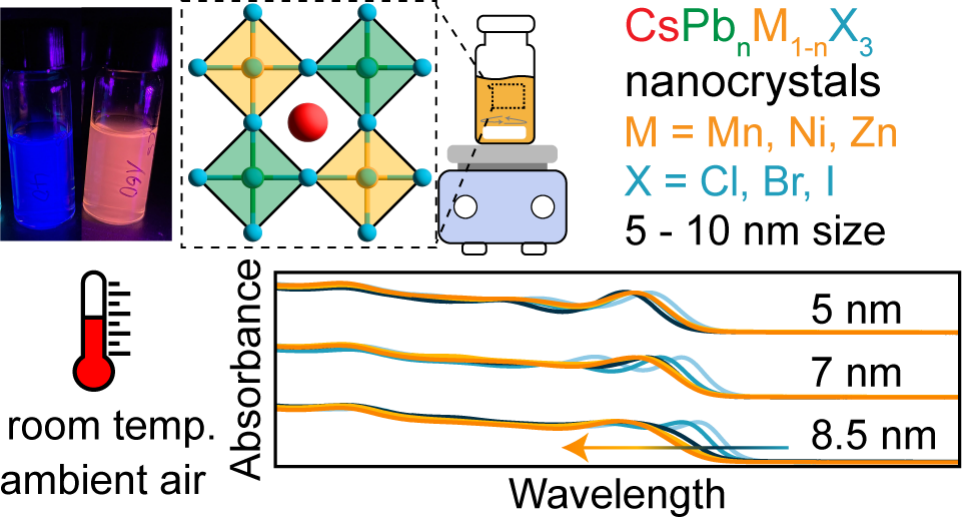

Halide perovskite nanocrystals are promising materials for optoelectronic applications. Metal doping provides an avenue to boost their performance further, e.g., by enhancing light emission, or to provide additional functionalities, such as nanoscale magnetism and polarization control. However, the synthesis of widely size-tunable nanocrystals with controlled doping levels has been inaccessible using traditional hot injection synthesis, preventing systematic studies on dopant effects toward device applications. Here, we report a versatile synthesis method for metal-doped perovskite nanocrystals with precise control over size and doping concentration under ambient conditions. Our room temperature approach results in fully size-tunable isovalent doping of CsPbX_3_ nanocrystals (X = Cl, Br, I) with various transition metals M^2+^ tested (M = Mn, Ni, Zn). This gives for the first time access to small, yet precisely doped quantum dots beyond the weak confinement regime reported so far. It also enables a comparative study of the photophysics across multiple size and dopant regimes, where we show dopant-induced localization to dominate over quantum confinement effects. This generalizable, facile synthesis method thus provides a toolbox for engineering perovskite nanocrystals toward light-emitting technologies under industrially relevant conditions.

## Introduction

The favorable optoelectronic properties of halide perovskite nanocrystals (NCs), such as their energetic tunability,^1,2^ fast radiative rates,^3^ and high photoluminescence quantum yields (PLQYs)^4^ make them attractive for a variety of optoelectronic applications.^5^ Those include light-emitting diodes,^6^ lasers,^7^ photovoltaics,^8^ photodetectors,^9^ photocatalysis,^10,11^ and quantum light sources.^12−14^ In general, halide perovskites are of ABX_3_ composition, where A is a small monovalent cation, such as Cs^+^, B is a divalent metal cation, most commonly Pb^2+^, and X is a halide, (Cl^–^, Br^–^, or I^–^). Substituting small quantities of metal ions in the B-site position can dramatically improve the already outstanding optoelectronic properties of the native NC or can endow new functionalities.^15−18^ For example, divalent metal dopants Zn^2+^, Mn^2+^, and Ni^2+^ have been previously shown to enhance the radiative recombination rate due to dopant-element specific hybridization with the host lattice, or element-unspecific lattice-periodicity breaking effects.^19,20^

However, so far only larger, weakly or unconfined metal-doped perovskite NCs have been reported, while the role of quantum confinement in the modification of the electronic and optical properties in doped NCs remains largely unclear. The main cause of this knowledge gap is the difficulty of synthesizing monodisperse, strongly confined NCs using conventional hot injection synthetic methods. Here, the reaction temperature impacts the resultant NC size, but the highly ionic nature of halide perovskites results in a much more rapid reaction compared to more covalently bound II–VI or III–V NCs, and thus the nucleation and growth processes cannot be temporally separated.^1,21^ Further, strongly confined NCs cannot be synthesized by simply lowering the temperature, as <140 °C, mixtures of cubic NCs and nanoplatelets can be observed.^22^ Recently, strongly confined undoped NCs have been reported and obtained either directly via precursor modification^23,24^ or through postsynthetic purification processes.^25^ To this point, no generalizable synthesis method exists for doped NCs that allows for the same size control and NC monodispersity as their undoped counterparts. Thus, dopant effects on the electronic properties of the host NC cannot be disentangled from quantum confinement effects.

Here, we describe such a method, resulting in size-tunable NCs doped with highly controlled dopant concentrations of various metals tested (Mn^2+^, Ni^2+^, or Zn^2+^), working under ambient conditions at room temperature. We validate and quantify lattice doping using electron paramagnetic resonance (EPR), the dopant-induced bandgap widening, and inductively coupled plasma mass spectrometry (ICP-MS). Using time-resolved optical spectroscopy, we study the now accessible series of NCs with systematically varied size and doping concentration and find surprisingly that the doping-induced carrier dynamics modulation persists independent of the level of quantum confinement. Further, the developed method is simple, scalable, and can be performed at room temperature, paving the way for future technological relevance.

## Results and Discussion

**Figure 1:**
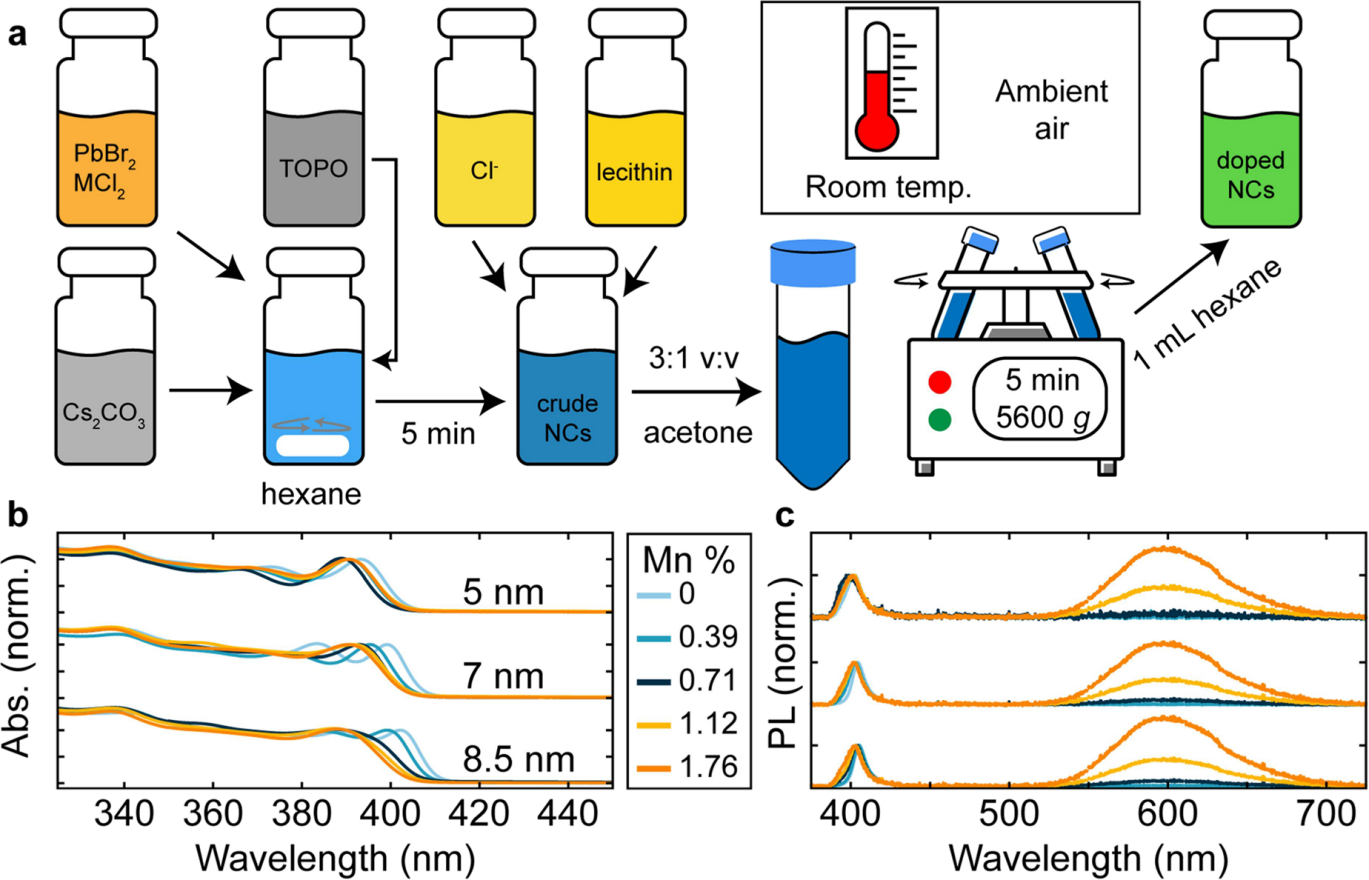
A synthetic access to size- and composition-tunable metal-doped halide perovskite nanocrystals (NCs) under ambient conditions. **a**, Schematic for the synthesis of metal (M) doped NCs. **b**, Normalized absorption spectra of CsPbCl_3_ NCs of varying size and Mn content. The legend shows the Mn concentration determined by inductively coupled plasma mass spectrometry for 8.5 nm NCs. **c**, Photoluminescence (PL) spectra of the same NCs shown in **b**. PL spectra were normalized to the excitonic perovskite peak in all cases, and samples were excited by a 365 nm continuous wave LED.

Metal-doped nanocrystals were synthesized according to the framework shown schematically in Figure 1a, building on the excellent work by Kovalenko and co-workers.^24^ Rather than a hot injection synthesis, where the NC size is dictated by the reaction temperature, this approach is governed by a reaction equilibrium between Cs^+^ and [PbX_3_]^−^ ion pairs, complexed to a neutral coordinating agent, trioctylphosphine oxide (TOPO). The injection of a Cs-diisooctylphosphinate (DOPA) solution into a solution containing PbBr_2_ and TOPO leads to the rapid formation of [PbBr_3_]^−^ anions, resulting in the ultimate conversion of Cs[PbBr_3_] complexes into CsPbBr_3_ NCs. The weakly coordinating TOPO can then be displaced by stronger binding ligands, such as the zwitterionic lecithin.^4,24^ The NC size is then governed by either stoichiometry or reaction time, where the concentration of TOPO is a convenient handle on the resultant NC size. We now partially substitute PbBr_2_ for dopant salts MCl_2_ (M = Mn^2+^, Ni^2+^, or Zn^2+^), e.g., in mole ratios of 95:5, 90:10, 75:25, and 50:50, and find that the NC synthesis proceeds as previously described. In a usual synthesis (see Methods Section for details), a 2:1 (v:v) ratio of Pb:MX_2_/TOPO and Cs/DOPA solutions were added to 6 mL of hexane in the presence of additional TOPO, where the amount added changed the NC size. In order to directly compare undoped and doped NCs with identical halide composition (not limiting ourselves to the initially resulting Br:Cl mixture), we employed different halide-shifting agents to exchange bromide anions for chloride anions, through the addition of oleylammonium chloride^2^ or a WCl_6_-TOPO complex at the end of the synthesis (Figure S1). While recent work by Akkerman and co-workers has relied on divalent metal salts as halide exchange agents, we chose organic routes or large metal salts with higher valencies to avoid potential uncontrolled, additional metal doping or cation exchange.^26,27^ Lecithin was then added as the surface ligand species, before typical purification methods, where a 3:1 v:v ratio of acetone was added before centrifugation, and the resultant NCs were redispersed in ∼ 1 mL of hexane, and passed through a syringe filter. We also emphasize that all syntheses we report here were performed under ambient conditions (i.e., on the benchtop with oxygen and moisture present) and at room temperature, making this method widely applicable, and meeting industry standards. First, taking Mn-doped NCs as an example case (we later show the generalizability to other dopants), we were able to synthesize size-tunable doped nanocrystals using the above-described mole ratios and various amounts of TOPO added to precisely tune the Mn concentration and NC size, respectively.

Upon the addition of Mn, the pure-chloride-doped NCs feature the expected pronounced blue shift in their excitonic absorption (Figure 1b) and photoluminescence (PL) (Figure 1c) compared to their undoped equivalents.^28−31^ This dopant-induced bandgap widening, which occurs in addition to that due to the bromide-to-chloride shift and the quantum confinement for a given size, was previously attributed to local NC lattice periodicity breaking caused by the electronic perturbation of the dopant.^19,20^ To verify that all doped and undoped NCs are indeed the same size, transmission electron microscopy (TEM) was performed for various sizes, resulting in similar mean NC edge lengths for doped and undoped NCs (Figures S2 and S3). Further, the Mn-dopant concentration in the final NC products was verified using ICP-MS for a variety of Mn-dopant feed ratios and NC sizes (Table S1). We note that reported Mn content is likely an upper bound, due to the difficulty of removing unreacted Mn from NC solutions.^32^ An excess of Mn is necessary for the synthesis of Mn-doped NCs, in line with previously reported synthetic routes.^32,33^ Interestingly, two doping regimes are observed, where for Pb:Mn feed ratios with less Mn (95:5 and 90:10), the final Mn concentration (relative to Pb) depends on the NC size, with larger NCs incorporating relatively more Mn. At higher Mn feed ratios (75:25 and 50:50), the Mn concentration is size-independent, resulting in a high maximal doping concentration of ∼ 1.8 atomic-% at a 50:50 Pb:Mn feed ratio. We note that the ICP-MS was performed after anion exchange, where some Mn may be expelled from the NC during the anion exchange.^34^ Careful observation of the smaller NC sizes in the absorption spectra in Figure 1b yields a slight red shift upon increasing Mn concentration. This is a size effect, where for small NC sizes and relatively high Mn concentrations, the mean NC size was relatively larger than the undoped NC (Figure S4). In concert with a general blue shift in the excitonic NC PL upon doping (Figure 1c), a broad emission can be observed around 600 nm, increasing in intensity relative to the excitonic PL for higher concentrations of Mn. This additional PL peak is attributed to the spin- and Laporte-forbidden Mn^2+^ ^4^T_1g_ → ^6^A_1g_ d-d emission, following energy transfer from the host NC to the dopant.^35−37^

When the halide anion is not shifted during synthesis, the host excitonic absorption and emission blue shift with higher Mn feed ratios due to a higher Cl content brought in, rather than the impact of the metal dopant (Figure S5). In the not fully halide-shifted case (*i.e*., largely bromide-rich Mn-doped CsPbX_3_ with only small amounts of chloride content), relatively weak Mn PL can still be observed, although it should be noted that CsPbX_3_ NCs with relatively higher Br content typically feature brighter PLQYs than their Cl heavy counterparts.^1,38^ Further, attempts to directly synthesize Mn-doped CsPbBr_3_ NCs using a 50:50 ratio of PbBr_2_:MnBr_2_ resulted in no optical blueshift and no observable Mn emission (Figure S6), highlighting the importance of chloride anions to the metal doping process, in line with previous results.^34^

**Figure 2:**
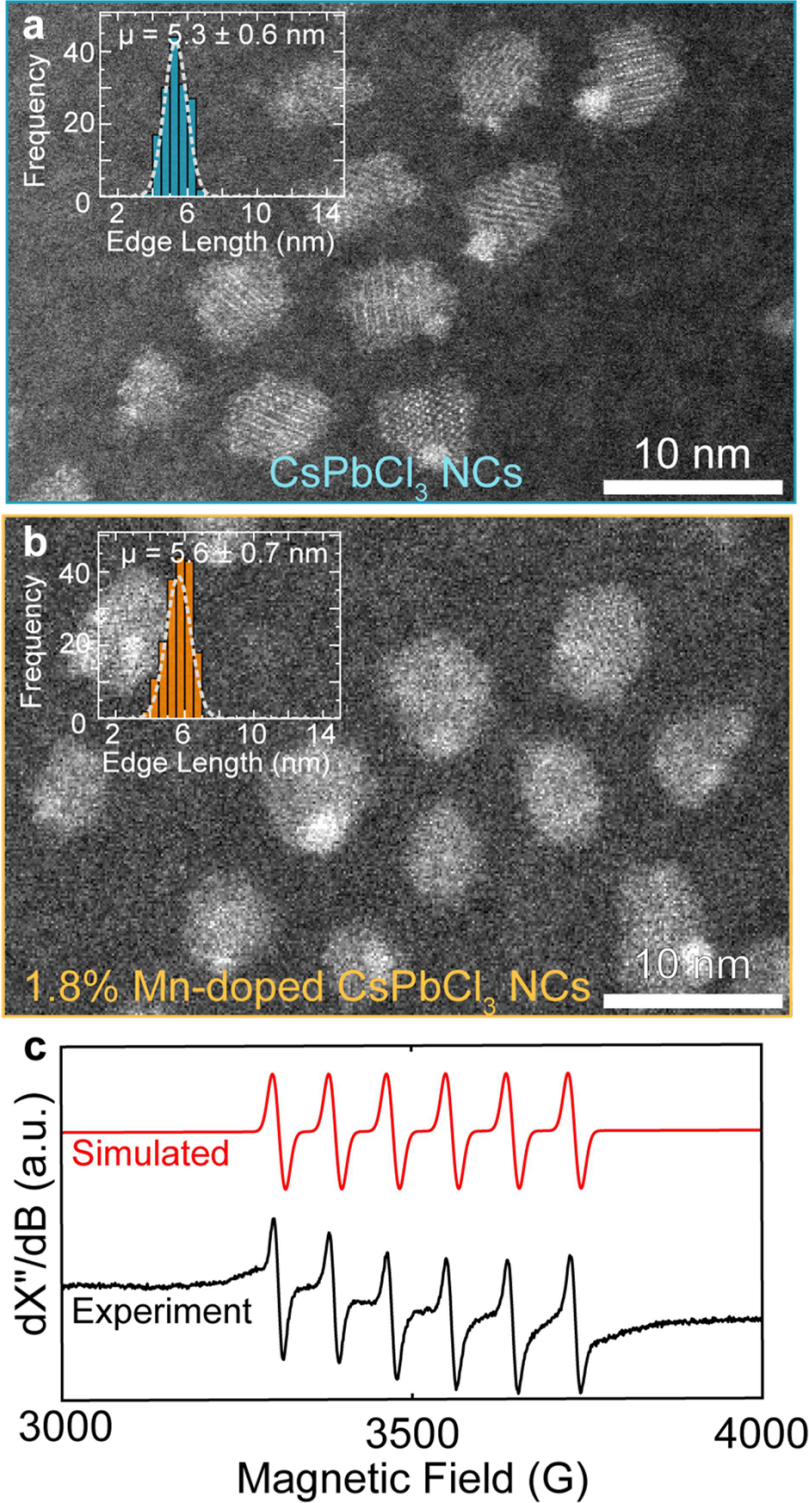
Evidence of metal dopant incorporation into nanocrystal lattice without influencing size. Exemplary HAADF-STEM micrographs of **a**, CsPbCl_3_ NCs, and **b**, 1.8% Mn-doped (by atom) CsPbCl_3_ NCs. Insets show size distributions and the resultant mean size and standard deviation, confirming no significant size changes upon doping, even for small and confined NCs. **c**, Simulated (red line, top) and experimental (black line, bottom) X-band electron paramagnetic resonance data for 1.8% Mn-doped CsPbCl_3_ NCs, recorded at room temperature, confirming dopant incorporation into the host crystal lattice.

The NC size and shape remain again largely unchanged upon doping, as shown by high-angle annular dark-field scanning TEM (HAADF-STEM) (Figure 2a,b), confirming that the blue shift observed in Figure 1b is not correlated with the NC size, but due to the dopant.

Additionally, X-band EPR of 1.8% Mn-doped NCs (Figure 2c) yielded a signal at *g* ≈ 2 with the expected splitting pattern of an S = 1/2 system coupled to a ^55^ Mn (I = 5/2) nucleus. Simulation with EasySpin^39^ yielded a hyperfine coupling constant of ^55^ Mn(A) = 85 G (or 238 MHz), which is similar to the hyperfine coupling constant observed in bulk Mn-doped CsPbCl_3_ (87 G),^40^ signifying that the Mn incorporates into the NC lattice. EPR of unshifted Mn-doped CsPb(Br/Cl)_3_ also yields the expected Mn hyperfine splitting pattern, with a slight background from magnetically concentrated Mn (Figure S7).^41^ X-ray diffraction patterns (Figure S8) of the synthesized undoped, Mn-doped, Ni-doped, and Zn-doped NCs match previously observed patterns for orthorhombic CsPbCl_3_ NCs.^42^ No significant differences in peak position between undoped and metal-doped NCs are observed. This is in line with previous work with similar metal-doping concentrations, where the small dopant concentration has largely negligible effects on the average lattice spacing, despite the smaller ionic radii of Mn, Ni, and Zn as compared to Pb.^32,43^

The ability to synthesize precisely size- and doping-level-tunable NCs allows for the elucidation of their photophysical properties systematically, decoupling effects due to dopant incorporation or halide composition from those of quantum confinement. The dynamics of charge recombination and energy transfer to Mn centers in Mn-doped CsPbX_3_ NCs have been previously studied through a combination of time-resolved optical spectroscopies, providing insights on carrier trapping, radiative recombination, energy/charge transfer to Mn, and Auger-like processes.^43−49^ So far, the inability to grow widely size-tunable Mn-doped NCs, where the NC size is not greatly influenced by the Mn-dopant, and with the inclusion of precisely tunable doping levels, has made it difficult to gain mechanistic insights in a systematic fashion.

**Figure 3:**
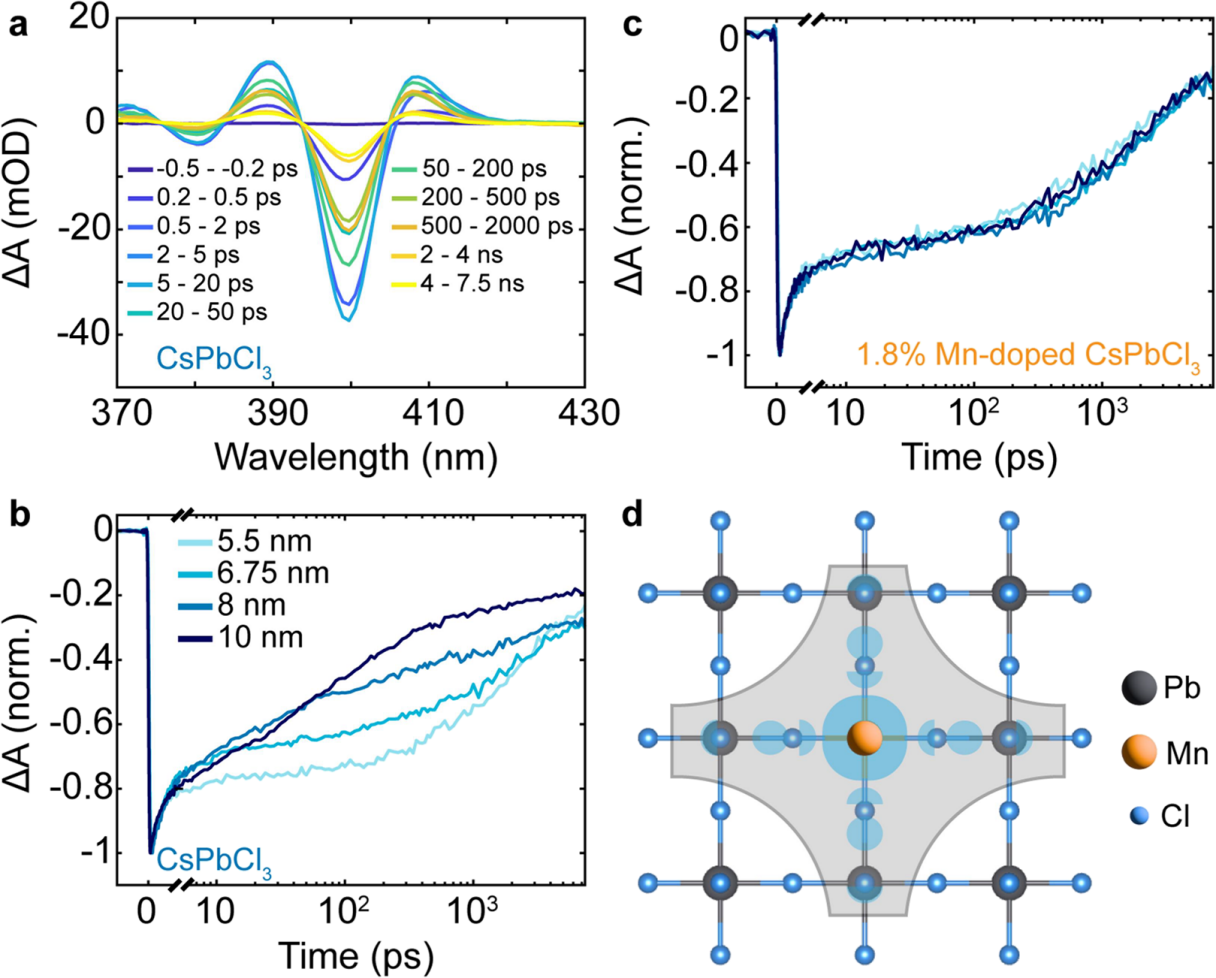
Excitation dynamics of doped nanocrystals as a function of quantum confinement. **a**, Transient absorption (TA) spectra of 5.5 nm CsPbCl_3_ NCs at various pump–probe delay times. **b**, Normalized TA kinetics probed at the respective ground-state bleach for CsPbCl_3_ NCs of different sizes. Faster recombination is observed for larger NCs **c**, Normalized TA bleach kinetics of 1.8 atom-% Mn-doped CsPbCl_3_ NCs for different sizes. No size dependence of the kinetics for doped samples is observed. All TA experiments were performed at a pump wavelength of 343 nm (ca. 100 fs pulses) with an excitation fluence of 3 μJ/cm^2^. **d**, Schematic of the electron density (shaded area) in a Mn-doped CsPbCl_3_ lattice, showing enhanced transient charge density around the dopant.

Figure 3a shows a representative set of transient absorption (TA) spectra for a CsPbCl_3_ NC ensemble in solution for various pump–probe delays, excited at 343 nm (ca. 100 fs long pulses). Further, TA spectra of other NC sizes for undoped NCs are shown in Figure S9, and for CsPbCl_3_ NCs doped with 1.8% Mn in Figure S10. In all spectra, three dominant features can be observed: (i) a negative feature corresponding to the excitonic ground state bleach (GSB), attributed to carrier filling upon excitation, (ii) a positive photoinduced absorption (PIA) feature blue-shifted from the GSB, and (iii) another PIA red-shifted from the GSB. The positive feature red-shifted from the GSB is commonly attributed to the presence of biexcitons.^50^ In our analysis, we focus on the ground-state exciton bleach and plot the kinetics at the respective maximum for each undoped NC size in Figure 3b.

We observe largely multiexponential appearing kinetics for all cases as expected, previously reported widely in the literature,^50−53^ which can be understood from the ensemble character of the nanocrystals having a distribution of respective sizes and trap densities (e.g., a different propensity of surface traps between two NCs in the solution). Therefore, we refrain here from the unphysical assignment of lifetimes from multiexponential fits to specific processes but note that generally charge trapping has been assigned to occur often on few-ps time scales,^43,54,55^ energy-transfer from host to dopant on a few-hundred ps time scale,^27,43,55^ radiative recombination on the sub-ns to few-ns time scale,^43,55,56^ and delayed trap-assisted recombination on the order of hundreds of ns.^19^ As the excitation density per NC, ⟨*N*⟩, is relatively low in the TA analysis reported here (<0.01 in all cases), we can largely rule out the presence of multiexcitonic species in the TA kinetics.^50,57^ Following this, the initially similar drop within the first few ps for all sizes of undoped NCs (Figure 3b) implies a similar amount of charge trapping and thus (surface-) trap density for all sizes produced. Similarly, the late-time decay tails assigned to trap-assisted recombination at the latest times remain largely unaffected by the NC size. However, the major decay component covering the range from tens of ps to about 1 ns varies strongly across the sizes and becomes faster for larger NCs. As this time scale corresponds mostly to intrinsic radiative recombination, we expect this trend to be a consequence of the “giant oscillator strength effect”, meaning that upon entering the weak confinement regime, the radiative lifetime dramatically shortens.^3,58^

When next turning to a series of 1.8% Mn-doped CsPbCl_3_ NCs of various sizes, we find the kinetics to change dramatically (Figure 3c). Very interestingly, we see near-identical kinetics for the various NC sizes upon doping. This “pinning” of kinetics, which to the best of our knowledge has not been observed before (due to the absence of a synthesis method to produce such a series so far), indicates a rate-determining step due to the presence of the dopant which surpasses the effect of quantum confinement, or its absence. We relate this observation again to the lattice-periodicity breaking of the dopant, an effective perturbation that prevents the delocalization of the exciton wave function across the whole nanocrystal volume, and also its modulation due to the quantum size effect (Figure 3d). Additionally, the TA ground-state exciton bleach kinetics for three NC sizes with varying Mn content is shown in Figure S11 in order to demonstrate the effect of lattice periodicity breaking effects with gradually increasing effective Mn doping levels. From the NC edge length and Mn concentration determined using ICP-MS, the effective doping level (i.e., # of Mn per NC, Mn density, or average Mn–Mn distance) can be calculated (see Supplemental note 1). These quantities are tabulated for the three NC sizes investigated in Figure S11 in Tables S2, S3, and S4. In larger NCs (e.g., 8.5 nm edge length), a relatively large effective Mn doping level is observed, approaching 14 Mn dopants per NC, even for the lowest Mn concentration. Therefore, the TA kinetics reflect a strong doping-induced localization effect, in line with the kinetics observed in Figure 3c.

For the smallest NC size (5 nm edge length), a lower effective doping level is observed (1.6 dopants per NC). In the smaller dopant concentrations, therefore, the kinetics are largely unmodulated. For higher effective doping levels (approaching 13 dopants per NC), the kinetics are again strongly modulated in line with the kinetics shown in Figure 3c, as a consequence of the dopant-induced lattice periodicity breaking effects.

Further, we use TA to elucidate differences in the surface dynamics to monitor the effects of the different chloride-shifting agents. Figure S12 shows TA kinetics taken from the GSB maxima for undoped and 1.8% Mn-doped CsPbCl_3_ NCs chloride shifted with oleylammonium chloride or WCl_6_/TOPO. In both cases, the recovery lessened in the first 5 ps, the time scale typically attributed to rapid carrier trapping in CsPbCl_3_ NCs.^43^ Therefore, we attribute the slower bleach recovery to a higher degree of surface passivation when oleylammonium chloride is used. This higher degree of surface passivation is in line with previous work, where excess amines allow for tighter cationic ligand binding.^21^ It is important to note though, that the injection of oleylammonium chloride also forms additional species present in solution, which can be visible in the UV–vis absorbance (Figure S13). Similar species have been previously reported as 2D layered Pb-halide species formed under an excess of oleylamine.^59^ These species can be separated by employing slightly longer mixing times (after ligand addition) and by syringe filtering the NC solution. Therefore, the anion-shifting agent can be chosen to reflect the priority for ease in synthesis (WCl_6_/TOPO), or for a higher degree of surface passivation (oleylammonium chloride), based on application requirements.

**Figure 4:**
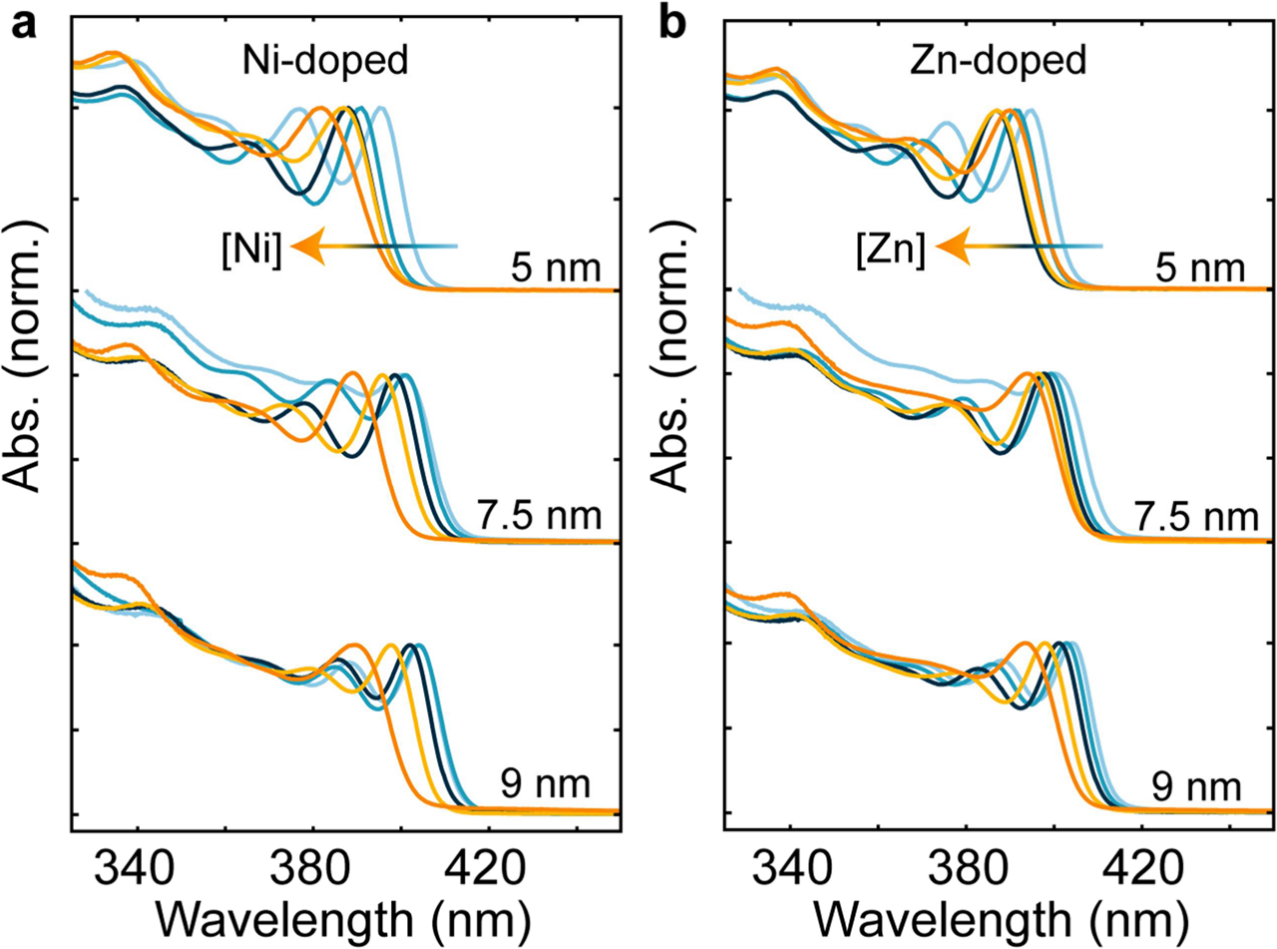
Generalizability of the nanocrystal doping method to other metals. **a**, Exemplary normalized absorption spectra of Ni, and **b**, of Zn-doped CsPbCl_3_ nanocrystals. In both cases, three different NC sizes are shown as a function of the respective dopant concentration, resulting in the expected dopant-induced blue shift due to lattice periodicity breaking for unchanged NC sizes (as confirmed with TEM) and halide composition.

The metal-doped NC synthesis route described here can also be extended to other dopants beyond manganese, as we show in the following. Figure 4 depicts the absorption spectra of CsPbCl_3_ NCs synthesized with the previously described molar ratios, but now employing either Ni^2+^ or Zn^2+^ dopants. For all three exemplary NC sizes shown in each case, the metal-doped NCs feature a distinct optical blue shift upon metal doping. Similarly to the Mn-doped case, this is not a size or halide composition effect, as the mean sizes of undoped and doped NCs are similar, as determined by TEM (Figure S14), and an excess of Cl^–^ is added in all cases to fully shift to a pure-chloride composition. This optical blue shift instead arises from the bandgap opening due to the same, element-unspecific lattice-periodicity breaking effect described before.^20^

Such alternative dopants are particularly exciting for light-emitting applications, as Ni and Zn can increase the radiative rate of these NCs and push their emission wavelength to the near-UV, especially when now combinable with the access to the strong confinement regime, but without featuring an additional decay channel toward the broad, slow orange additional emission as Mn does. Excitingly, in the case of Ni-doped CsPbCl_3_, the largest metal doping ratio (50:50 Ni:Pb feed ratio) for the smallest NC size (5 nm) does not result in relative red shifting compared to the next highest metal doping concentration, an effect attributed to a small size increase, observed in Figure S4 for Mn-doped CsPbCl_3_ NCs. The deep-UV nature of the optoelectronic properties of 5 nm Ni-doped CsPbCl_3_ NCs (peak absorption ∼ 382 nm) are in line with landmark UV LED materials with perovskite structures,^60^ setting the groundwork for use in optoelectronic devices.

The composition of metal-doped halide perovskite NCs can be further engineered by postsynthetic halide exchange to synthesize metal-doped CsPbBr_3_ and CsPbI_3_ NCs. Trimethylsilyl halide (TMS-X) reagents are convenient for this purpose,^61^ and have been recently employed in Mn-doped perovskite nanocrystals to engineer the NC composition without loss of Mn.^41^ The absorption and PL of three exemplary sizes of Mn-doped CsPbBr_3_ NCs shifted with an excess of TMS-Br are shown in Figure S15. Interestingly, in the smallest NCs (5 and 6.5 nm edge length), the blue-shift ascribed to lattice periodicity breaking effects gradually lessens upon larger Mn doping compositions (1.12 and 1.76% Mn), despite the observation of faint Mn emission. In previous work by Gamelin and co-workers, the addition of TMS-Br resulted in antiferromagnetic Mn clusters.^41^ The EPR spectrum of Mn-doped CsPbBr_3_ NCs, shown in Figure S16, also shows the disappearance of the hyperfine splitting pattern observed in Figure 2c upon halide exchange. Therefore, we hypothesize that persistent Mn clustering lessens or removes the effective lattice periodicity breaking, resulting in the gradual lessening of the optical blueshift in smaller NCs with relatively less Mn per NC. The Mn emission in CsPbBr_3_ NCs is also red-shifted relative to Mn PL observed in Mn-doped CsPbCl_3_ NCs (Figure S17), an observation that has been previously ascribed to Mn clustering or stronger Mn–Mn interactions.^62^ Further, a large excess of TMS-I was added to obtain Mn-doped CsPbI_3_ NCs. The absorption and PL of Mn-doped CsPbI_3_ NCs are shown in Figure S18. The smallest NCs (5 nm) and NCs with relatively higher Mn content (1.12 and 1.76%) rapidly degraded before further analysis. Interestingly, the optical blueshift is maintained in the absorption and PL, suggesting that Mn remains incorporated. No Mn PL is observed, as energy transfer to Mn is energetically unfavorable.^34^

A generalizable synthesis route for metal-doped halide perovskite NCs is described, which enables full size- and composition tunability, which is scalable and operates under ambient conditions at room temperature. This method incorporates metal dopants into the lattice during synthesis, as confirmed by EPR and the dopant-induced bandgap blue shift. Further, this method allows for unprecedented synthetic control across multiple NC size, halide composition, and dopant level regimes, allowing for the first systematic comparative study of the size-dependent photophysical properties of metal-doped NCs. Ultrafast spectroscopy revealed that the dopant-induced excitation localization is NC size-independent for the compositions studied.

The described synthesis method thus lays the groundwork for halide perovskite NCs to be finally studied with respect to their fundamental properties in a *systematic* fashion, varying one parameter at a time (size, halide composition and doping level). This will be necessary to answer open questions, *e.g*., about the dark/bright nature of the energy levels (and their ordering), and how they give rise to the strong luminescence observed in these materials. Of particular current interest for device performance is the deep-blue to UV spectral region of light-emitting diodes, for use in displays and water disinfection. Here, doping will provide an additional lever to further blue-shift the bandgap while sustaining stability. Importantly, controlled metal doping may also enable new functionalities, such as through magnetic exchange interactions in the case of magnetic dopants for hitherto unexplored applications in spin-photonic interfaces and quantum information processing. Lastly, we are hopeful that the simplicity, scalability and ambient working conditions of this method will pave the way for technology transfer to the industrial level.

## Supporting Information Available

Experimental methods and additional experimental data, including steady-state absorption and photoluminescence, transmission electron micrographs, X-ray diffraction, and transient absorption (PDF)
