# Supplementary material for: Metal Doping of Strongly Confined Halide Perovskite Nanocrystals under Ambient Conditions
Source: J Am Chem Soc. 2025 May 5;147(19):16536–44. doi: 10.1021/jacs.5c03629 (PMC12082692; doi:10.1021/jacs.5c03629)
Supplement: Supplementary file 1 — ja5c03629_si_001.pdf [file ja5c03629_si_001.pdf]

Supporting Information

Metal doping of strongly confined halide perovskite nanocrystals under ambient conditions

Zachary A. VanOrman,[1,2] Mateo Cárdenes Wuttig,[1] Antti-Pekka M. Reponen,[1] Taek-Seung Kim,[1] Claire E. Casaday,[3] Dongtao Cui,[3] Tejas Deshpande,[2] Huygen J. Jöbsis,[2] Pascal Schouwink,[4] Emad Oveisi,[5] Aurélien Bornet,[2] Christian Reece,[1] and Sascha Feldmann[1,2]*

[1]*Rowland Institute, Harvard University, Cambridge, MA, 02142, USA.*
[2]*Institute of Chemical Sciences and Engineering, École Polytechnique Fédérale de Lausanne, Lausanne, 1015, Switzerland.*
[3]*Department of Chemistry and Chemical Biology, Harvard University, Cambridge, MA, 02138, USA.*
[4]*X-ray Diffraction and Surface Analytics Platform, École Polytechnique Fédérale de Lausanne, Sion, 1950, Switzerland.*
[5]*Interdisciplinary Centre for Electron Microscopy (CIME), École Polytechnique Fédérale de Lausanne, Lausanne, 1015, Switzerland.*

*sascha.feldmann@epfl.ch

Experimental methods

Oleylammonium chloride synthesis

All reagents were used as received unless otherwise stated. Oleylammonium chloride was synthesized using the method described by Kovalenko and coworkers.[1] Briefly, 0.038 mol of oleylamine (70% technical grade, Sigma Aldrich) was added to 100 mL of ethanol (99.5%, VWR Chemicals) in an ice bath, and 0.076 mol of HCl (37%, Sigma Aldrich) was added dropwise. The solution was left to stir overnight. The solvent was evaporated, resulting in a yellow-white solid that was rinsed multiple times with diethyl ether before drying overnight on a vacuum line, yielding a white powder.

Stock solution preparation

Pb:MX_2 (M = Mn, Ni, Zn) stock solutions were generally prepared by adding different mole ratios (100:0, 95:5, 90:10, 75:25, 50:50) of lead bromide (99.999%, Sigma Aldrich) to either manganese chloride (99.999%, Sigma Aldrich), nickel chloride (99.99%, Sigma Aldrich) or zinc chloride (99.99%, Sigma Aldrich), where the total mole ratio equaled 0.5 mmol of combined metal salt, into a vial with 2.5 mL of octane (99%, Thermo Scientific) and 2.5 mmol of tricoctylphosphine oxide (TOPO, 90%, Sigma Aldrich). The vials were heated to 120 °C for 1 hour, and upon cooling, 10 mL of hexane (95%, Thermo Scientific) was added, and the solution was syringe-filtered.

The Cs_2CO_3 stock solution was prepared by adding 100 mg of Cs_2CO_3 (99.9%, Sigma Aldrich) into a vial containing 1 mL of diisooctylphosphinic acid (DOPA, 90%, Sigma Aldrich) and 2 mL of octane. The vial was heated to 120 °C for 1 hour, and upon cooling, 27 mL of hexane was added, and the solution was syringe-filtered.

The TOPO stock solution was prepared by adding 2.32 g of TOPO into a vial with 30 mL of hexane. The solution was mixed until fully dissolved and syringe filtered.

The oleylammonium chloride stock solution was prepared by dissolving oleylammonium chloride into toluene (99.5%, Thermo Scientific) in a 10 mg/mL ratio.

The WCl_6 stock solution was prepared by adding 0.5 mmol of WCl_6 (99.9%, Sigma Aldrich) into a vial with 2.5 mL of octane and 2.5 mmol of TOPO. The vial was heated to 120 °C for 1 hour, and upon cooling, 10 mL of hexane was added, and the solution was syringe-filtered.

The lecithin stock solution was prepared by adding lecithin (90%, from soy, Thermo Scientific) to hexane in a 50 mg/mL ratio. The solution was mixed until fully dissolved and syringe filtered.

General $CsPbCl_3$ NC synthesis method

In general, 80 µL of a Pb:MX_2 stock solution (depending on desired metal dopant amount) and 120 – 360 µL of the TOPO stock solution were added to a vial containing 6 mL of hexane. 40 µL of the Cs_2CO_3 stock solution was added to initiate the reaction. After 4 minutes and 30 seconds, either 1 mL of oleylammonium chloride or WCl_6 solution was added, resulting in the solution becoming clear. At 5 minutes after the initiation of the synthesis, 40 µL of the lecithin stock solution was added. The solution would stir for another 5 minutes to aid in purification of layered 2D lead halide/oleylammonium materials, although no difference was observed when WCl_6 was used. The solution was transferred to a centrifuge tube, and a 3:1 v:v ratio of acetone (99%, VWR) was added to the tube. The NCs were then centrifuged at 5600 *g* (7480 rpm in this case) for 5 min. The NC pellets were dried for 5 minutes before redispersed in 1 mL hexane. To further purify the NC colloids, all synthesized NC solutions were passed through a 0.45 µm hydrophobic PTFE syringe filter before further analysis.

Post-synthetic halide exchange

Mn-doped $CsPbBr_3$ and $CsPbI_3$ NCs were obtained from $CsPbCl_3$ and $CsPbBr_3$ NCs, respectively, through post-synthetic halide exchange using trimethylsilyl halide (TMS-X) reagents,[2] TMS-Br and TMS-I, respectively. In a glovebox, a large excess (80 μL) of TMS-Br or TMS-I were added to purified NCs and allowed to react in a vial with a spinning stir bar for 30 minutes. The NCs were then collected for further analysis

Inductively coupled plasma mass spectrometry (ICP-MS)

ICP-MS was used to accurately determine the atomic-percent level of transition-metal doping, as its sensitivity well exceeds that of other methods (e.g. energy-dispersive X-ray spectroscopy, EDX). ~ 50 mg of as-synthesized Mn-doped NCs were digested in 10 mL of aqua regia using microwave digestion. The weight percentage of Pb and Mn were determined using an Agilent 7800 ICP-MS for 10 different samples.

Steady-state optical spectroscopy

UV-Vis absorption was measured using a Cary 5000 UV-Vis spectrophotometer with a spectral bandwidth of 2 nm. Steady-state photoluminescence was measured using a 365 nm LED (Thorlabs SOLIS-365C) as excitation source equipped with a 365 nm bandpass filter (Newport) and a CCD spectrometer (Thorlabs CCS200M). A 385 nm long pass filter (Newport) was placed after the sample to remove excitation light scatter.

Transient absorption (TA) spectroscopy

TA was measured using a previously described setup[3] based on a 1030 nm seed laser (PHAROS, Light Conversion, Yb:KGW lasing medium, 400 μJ pulse energy, 150 fs duration, 50 kHz repetition rate). The 343 nm pump beam (800 μm in diameter) was generated from the first and second harmonics of the 1030 nm seed laser using a harmonic generation unit equipped with nonlinear crystals (beta-barium borate, lithium triborate). (HIRO, Light Conversion). The residual first and second harmonics were removed by dichroic mirrors. The third harmonic (343 nm) pump was passed through an optical chopper (100 Hz), where a portion of the pump was routed to a photodiode using a beam splitter in order to sort generated probe measurements into pumped and unpumped. The probe beam (200 μm in diameter) was generated from the second harmonic of the seed laser using quasi-supercontinuum generation in a sapphire crystal. The probe beam was passed into a grating spectrograph (Andor Kymera 193i) and recorded using a Si NMOS photodiode array detector (256 pixels). All TA experiments were conducted at pump fluences ~ 3 $\mu J/cm^2$. NC samples were measured in a 1 mm path-length quartz cuvette (Hellma) at concentrations ~ 0.5 μM.

Structural characterization

For transmission electron microscopy (TEM) imaging of samples, 10 μl of the as-synthesized NC solution was dropped onto the TEM grid (FCF300-CU-UB, Electron Microscopy Science) once using a micropipette. The morphologies of samples with histogrammed sizes were collected using a JEOL ARM 200F TEM at 200 kV.

Scanning transmission electron microscopy (STEM) images were acquired under high-angle annular dark-field (HAADF) conditions using a probe-corrected Thermo Fisher Scientific Spectra200 S/TEM operated at 200 kV with a beam current of 150 pA. This microscope is equipped with an ultra-high-brightness cold field emission gun (X-CFEG) and Velox acquisition software.

X-ray diffraction patterns (XRD) were collected in Bragg-Brentano geometry using a Bruker D8 Discover diffractometer equipped with a Cu X-ray source, a Johansson monochromator and LynxEye XE detector. The powders were spread out over low background Si sample holder in a way to achieve identical sample height for all samples.

Electron paramagnetic resonance (EPR)

X-band continuous-wave (CW) EPR spectra of $CsPbCl_3$ NCs were recorded on a Bruker ElexSys E500 spectrometer. All spectra were recorded at room temperature as suspensions in hexane. Spectrometer settings were as follows: frequency = 9.86 GHz, power = 0.6325 mW, gain = 20 dB, modulation amplitude = 8.0 G, modulation frequency = 100 kHz, 32 sweeps. All spectra were simulated using EasySpin[4] to obtain effective *g* values and hyperfine coupling constants.

X-band continuous-wave (CW) EPR spectra of $CsPb(Br/Cl)_3$ and $CsPbBr_3$ NCs were recorded on a Bruker Bruker EMX Nano benchtop spectrometer. All spectra were recorded at room temperature as suspensions in hexane. Spectrometer settings were as follows: frequency = 9.62 GHz, power = 0.3162 mW, gain = 40 dB, modulation amplitude = 4.0 G, modulation frequency = 100 kHz, 256 sweeps.

Supplemental notes:

Supplemental note 1: Calculation of Mn density per nanocrystal and Mn-Mn distance

The unit cell volume, V (in nm^3) of a $CsPbCl_3$ was calculated by cubing the unit cell parameter (a) of a bulk $CsPbCl_3$ perovskite.[5]

$$V = a^3 \tag{S1}$$

Assuming the NCs are cubic, the volume of a nanocrystal (V_{NC}) can be obtained from the edge length, L

$$V_{NC} = L^3 \tag{S2}$$

The number of unit cells can be obtained by dividing the NC volume by the unit cell volume,

$$\#\ of\ unit\ cells = \frac{V_{NC}}{V} \tag{S3}$$

As the Mn:Pb ratio was determined empirically using ICP-MS, and there is one Pb ion per unit cell, the number of Mn ions per nanocrystal can be determined by multiplying the Mn:Pb ratio by the number of Pb ions.

$$Mn\ ions\ per\ NC = Mn{:}Pb * \#\ of\ unit\ cells \tag{S4}$$

The Mn density (per nm^3), ρ_{Mn}, is obtained by dividing the Mn ions per nanocrystal by V_{NC},

$$\rho_{Mn} = \frac{Mn\ ions\ per\ NC}{V_{NC}} \tag{S5}$$

The average Mn-Mn distance (d_{Mn}), assuming a simple cubic approximation, the nearest neighbor Mn-Mn distance can be estimated by,

$$d_{Mn} \approx \rho_{Mn}^{-\frac{1}{3}} \tag{S6}$$

The ρ_{Mn} and d_{Mn} for relevant sizes are tabulated in Tables S2-S4.

Supplemental note 2: Calculation of average excitations per nanocrystal $\langle N \rangle$

The excitation fluence per pulse F (in $\mu J/cm^2$) can be calculated from the pulsed laser pump power, P, the laser repetition rate, R_{rep}, and the beam radius r_{ex} through:

$$F = \frac{P}{R_{rep}\pi r_{ex}^2} \tag{S7}$$

The photon fluence per pulse, j, in cm^{-2}, is given by:

$$j = \frac{P\lambda\pi r_{ex}^2}{R_{rep}hc} \tag{S8}$$

where λ corresponds to the pump wavelength, c is the speed of light in a vacuum, and h is Planck's constant. The absorbed photon density per pulse, j_{abs} is given by:

$$j_{abs} = j\frac{A}{l} \tag{S9}$$

where A corresponds to the absorbance of the sample in a cuvette with a path length l at the excitation wavelength.

The density of NCs, ρ_{NC} is related to the NC concentration c_{NC} (in mg/mL), the volume of the NC, V_{NC}, and the weight density of the material ρ_m by:

$$\rho_{NC} = \frac{c_{NC}}{V_{NC}\rho_m} \tag{S10}$$

The average excitations per NC, $\langle N \rangle$, can be calculated by:

$$\langle N \rangle = \frac{j_{abs}}{\rho_{NC}} \tag{S11}$$

Supplemental data

NC size (nm)	Pb:Mn feed ratio (mol %)	Weight-% Mn (relative to Pb)	Atomic-% Mn (relative to Pb)
5 nm	95:5	0.06 $\pm$ 0.0008	0.23 $\pm$ 0.0022
8.5 nm	95:5	0.10 $\pm$ 0.0001	0.39 $\pm$ 0.0003
5 nm	90:10	0.11 $\pm$ 0.0001	0.42 $\pm$ 0.0003
7 nm	90:10	0.19 $\pm$ 0.0005	0.71 $\pm$ 0.001
8.5 nm	90:10	0.19 $\pm$ 0.001	0.71 $\pm$ 0.003
5 nm	75:25	0.30 $\pm$ 0.001	1.12 $\pm$ 0.003
8.5 nm	75:25	0.30 $\pm$ 0.0004	1.12$\pm$ 0.0009
5 nm	50:50	0.48 $\pm$ 0.003	1.80 $\pm$ 0.008
7 nm	50:50	0.50 $\pm$ 0.001	1.89 $\pm$ 0.004
8.5 nm	50:50	0.47 $\pm$ 0.001	1.76 $\pm$ 0.003

Table S1: Inductively-coupled plasma mass spectrometry (ICP-MS) determined Mn atomic doping concentration relative to Pb, for $CsPbCl_3$ NCs with varying NC size (confirmed through TEM) and Pb:Mn feed ratio.

5 nm edge length $CsPbCl_3$ NC				
Mn:Pb ratio (%)	0.23	0.42	1.12	1.8
# of Mn per NC	1.6	3.0	8.0	12.8
ρ_{Mn} (nm^{-3})	0.013	0.024	0.064	0.102
d_{Mn} (nm)	4.3	3.5	2.5	2.1

Table S2: Mn:Pb ratio (determined from ICP-MS), and calculated values for # of Mn ions per nanocrystal, Mn density (ρ_{Mn}), and average Mn-Mn distance, d_{Mn} for a 5 nm edge length NC.

7 nm edge length $CsPbCl_3$ NC				
Mn:Pb ratio (%)	0.39	0.71	1.12	1.8
# of Mn per NC	7.6	13.8	21.8	35
ρ_{Mn} (nm^{-3})	0.061	0.11	0.18	0.28
d_{Mn} (nm)	2.5	2.1	1.8	1.5

Table S3: Mn:Pb ratio (determined from ICP-MS), and calculated values for # of Mn ions per nanocrystal, Mn density (ρ_{Mn}), and average Mn-Mn distance, d_{Mn} for a 7 nm edge length NC.

8.5 nm edge length $CsPbCl_3$ NC				
Mn:Pb ratio (%)	0.39	0.71	1.12	1.8
# of Mn per NC	13.6	24.8	39.1	62.8
ρ_{Mn} (nm^{-3})	0.11	0.20	0.31	0.50
d_{Mn} (nm)	2.1	1.7	1.5	1.3

Table S4: Mn:Pb ratio (determined from ICP-MS), and calculated values for # of Mn ions per nanocrystal, Mn density (ρ_{Mn}), and average Mn-Mn distance, d_{Mn} for an 8.5 nm edge length NC.

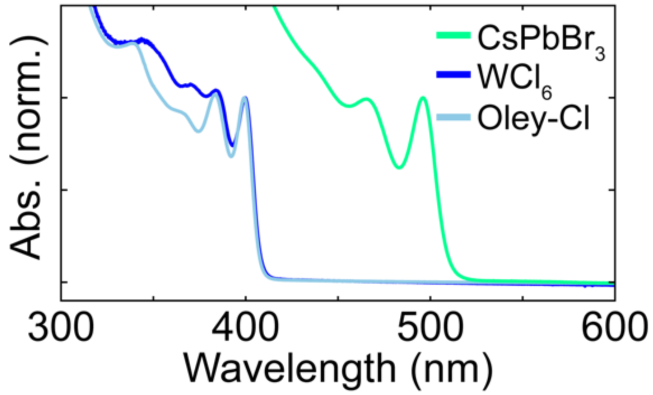

Fig. S1: Absorption spectra of as-synthesized 7 nm edge length $CsPbBr_3$ nanocrystals (green line), and $CsPbCl_3$ nanocrystals chloride-shifted from $CsPbBr_3$ using either 1 mL of WCl_6 (dark blue line) or oleylammonium chloride (light blue line) solution.

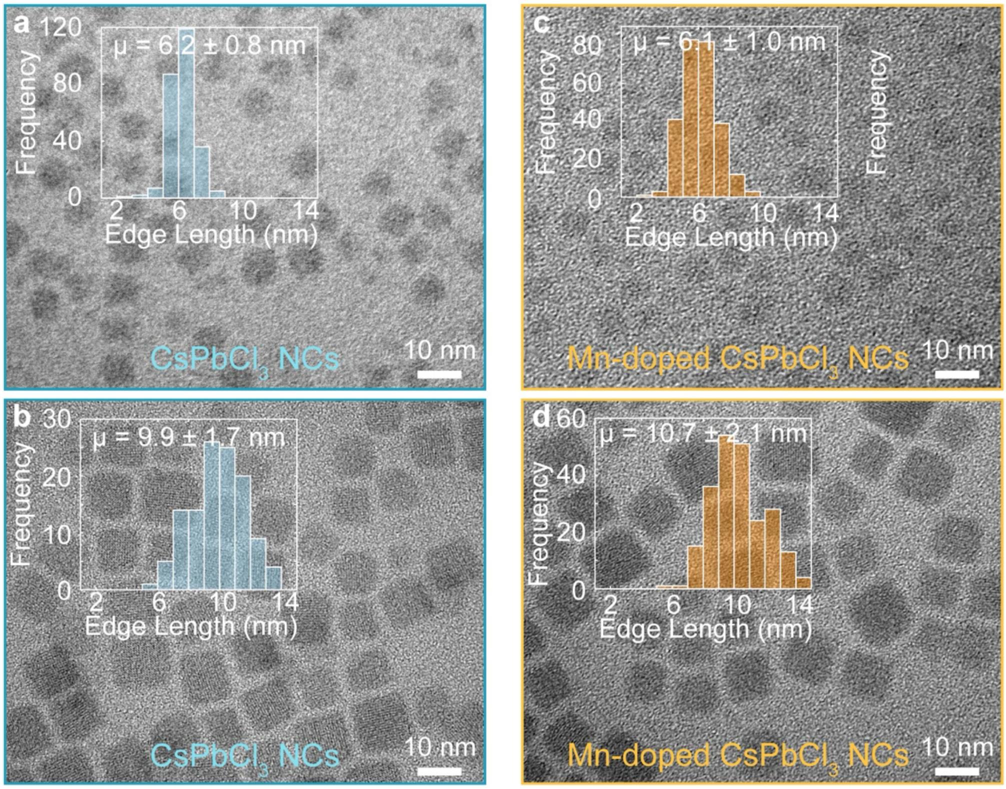

Fig. S2: TEM micrographs of (a) and (b) undoped $CsPbCl_3$ nanocrystals (NCs) and (c) and (d) 1.8% Mn-doped $CsPbCl_3$ NCs, respectively. (a) and (c) and (d) were synthesized under identical conditions, respectively, except for the addition of $MnCl_2$. In each case, a size histogram of multiple NCs, along with the mean size and standard deviation shown in the inset.

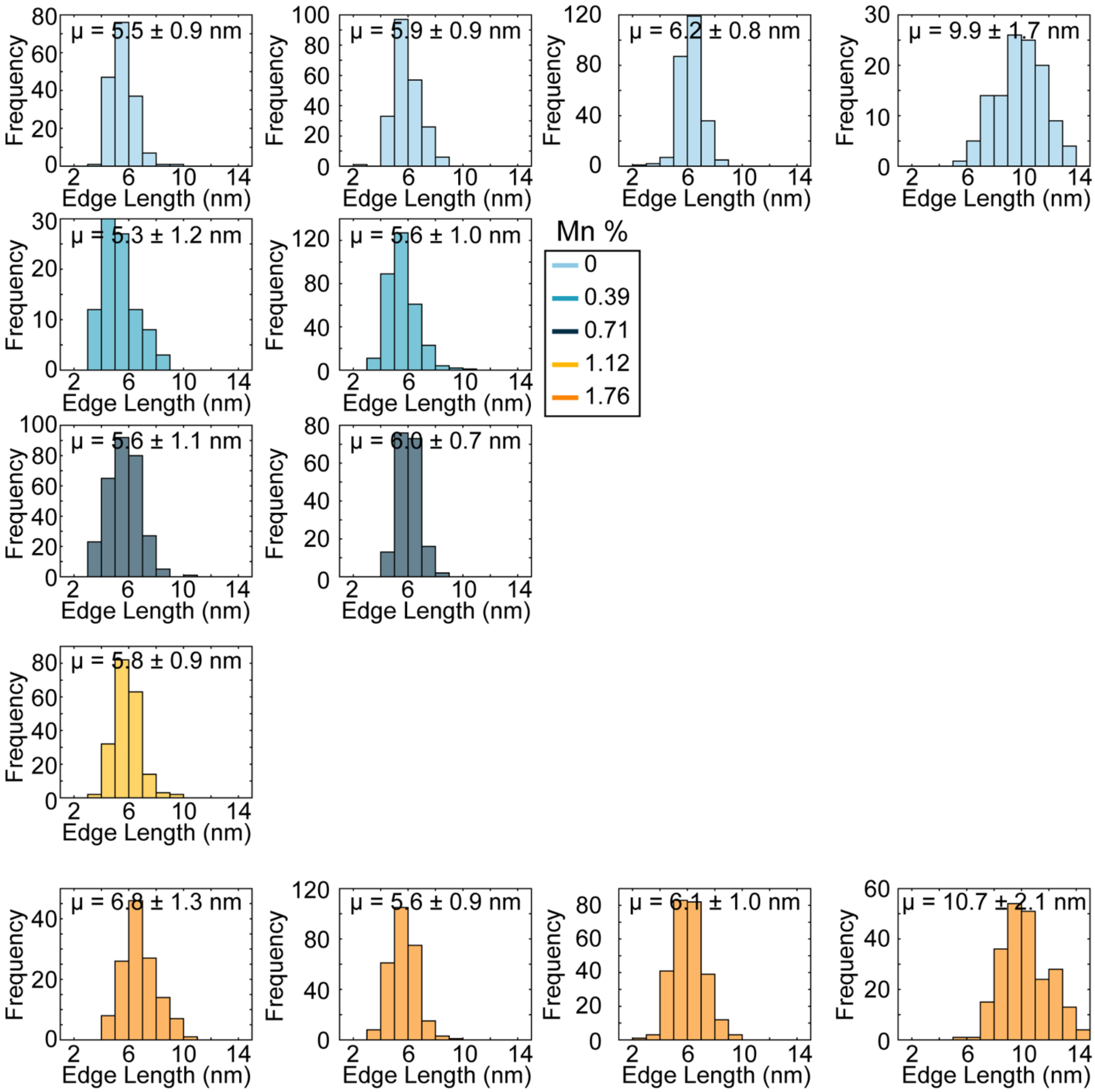

Fig. S3: Histogrammed sizes of $CsPbCl_3$ nanocrystals of varying size and Mn content determined from transmission electron micrographs. The Mn content for the largest size is given in the legend.

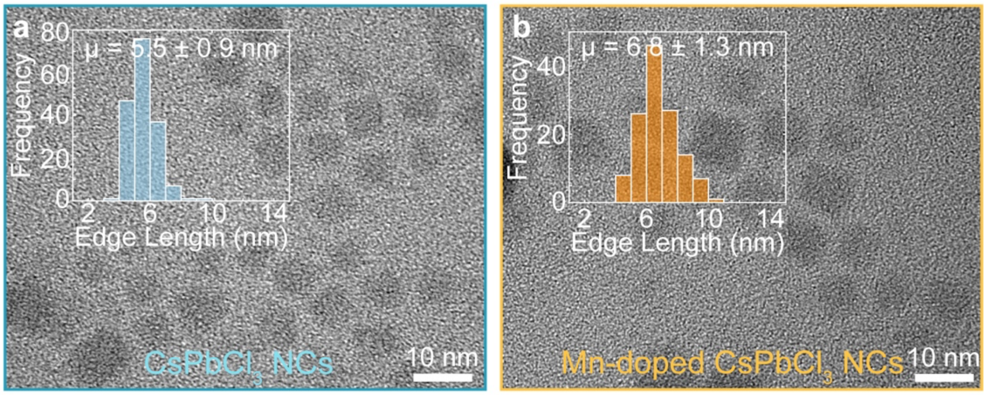

Fig. S4: TEM micrographs of (a) $CsPbCl_3$ nanocrystals (NCs) and (b) 1.8% Mn-doped $CsPbCl_3$ NCs synthesized under otherwise identical conditions. The inset shows histogrammed NC sizes with the mean size and standard deviation.

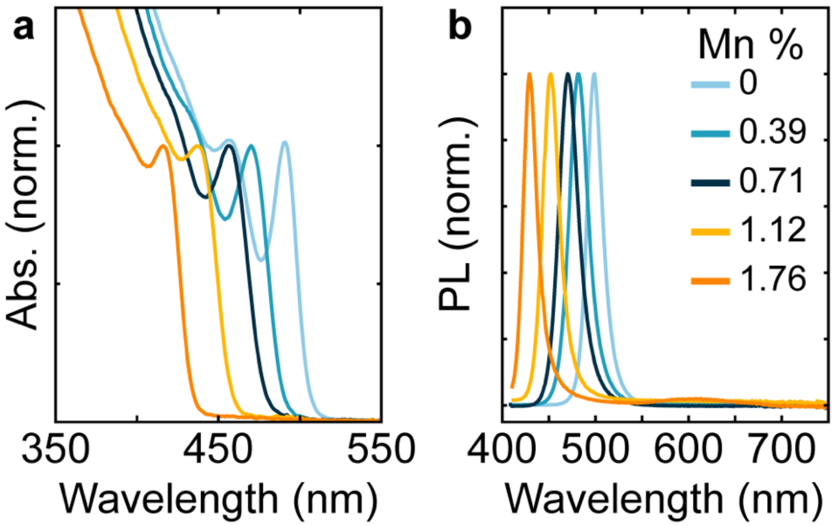

Fig. S5: Absorbance (a) and photoluminescence (b) of 6.5 nm edge length $CsPb(Br,Cl)_3$ nanocrystals with varying Mn dopant concentration.

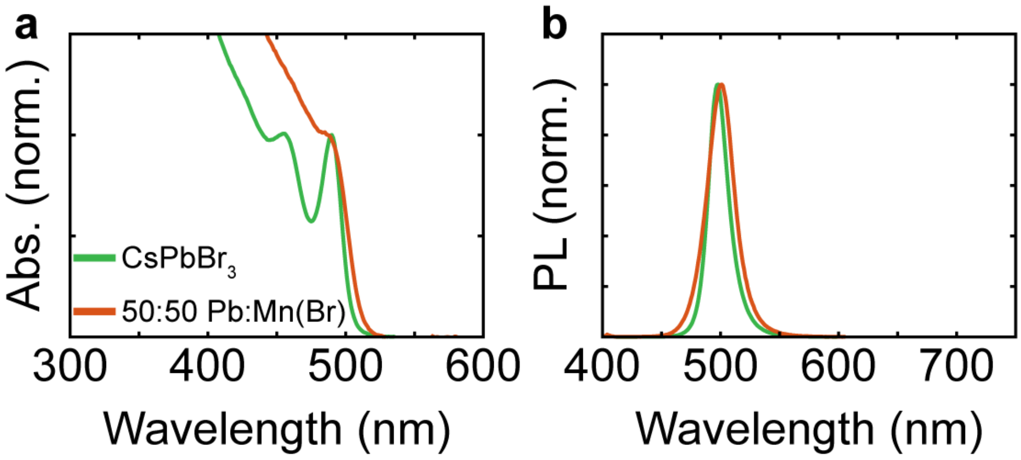

Fig. S6: (a) Absorbance and (b) photoluminescence of $CsPbBr_3$ nanocrystals synthesized with $PbBr_2$/TOPO stock solution (green) and a stock solution comprised of a 50:50 molar ratio of $PbBr_2$ and $MnBr_2$.

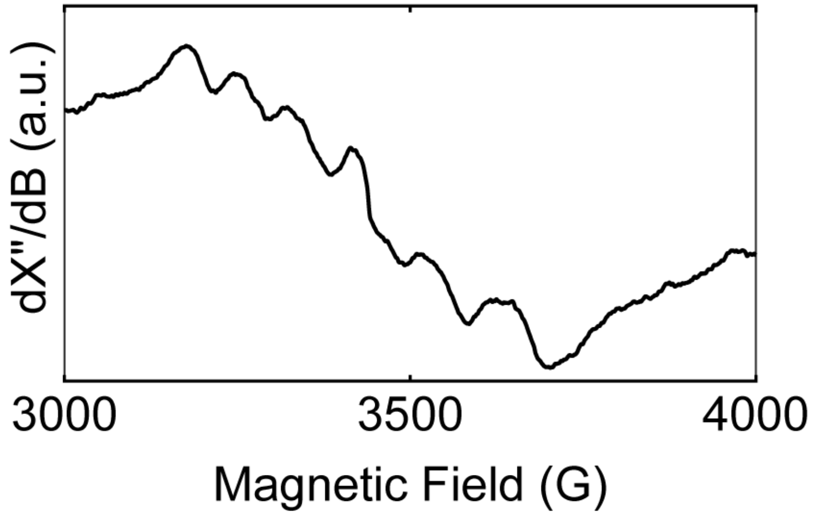

Fig. S7: X-band electron paramagnetic resonance data for Mn-doped $CsPb(Br_xCl_{1-x})_3$ nanocrystals recorded at room temperature.

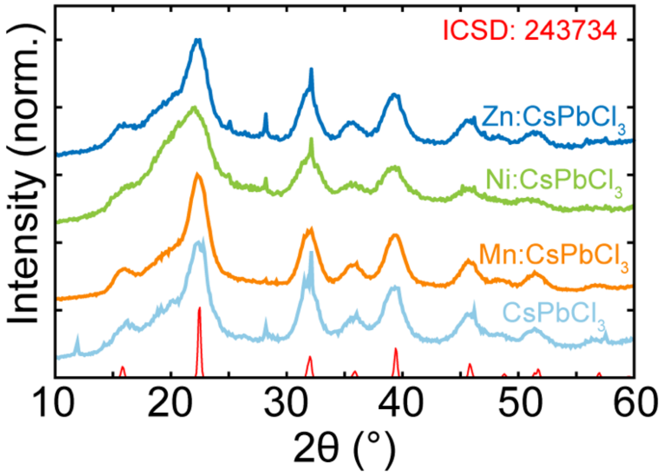

Fig. S8: Normalized X-ray diffraction patterns, vertically offset for clarity, of undoped $CsPbCl_3$ nanocrystals (light blue line), 1.8% Mn-doped $CsPbCl_3$ (orange line), Ni-doped $CsPbCl_3$ (green line), and Zn-doped $CsPbCl_3$. The XRD reference (red line) corresponds to $CsPbCl_3$ (ICSD 243734).

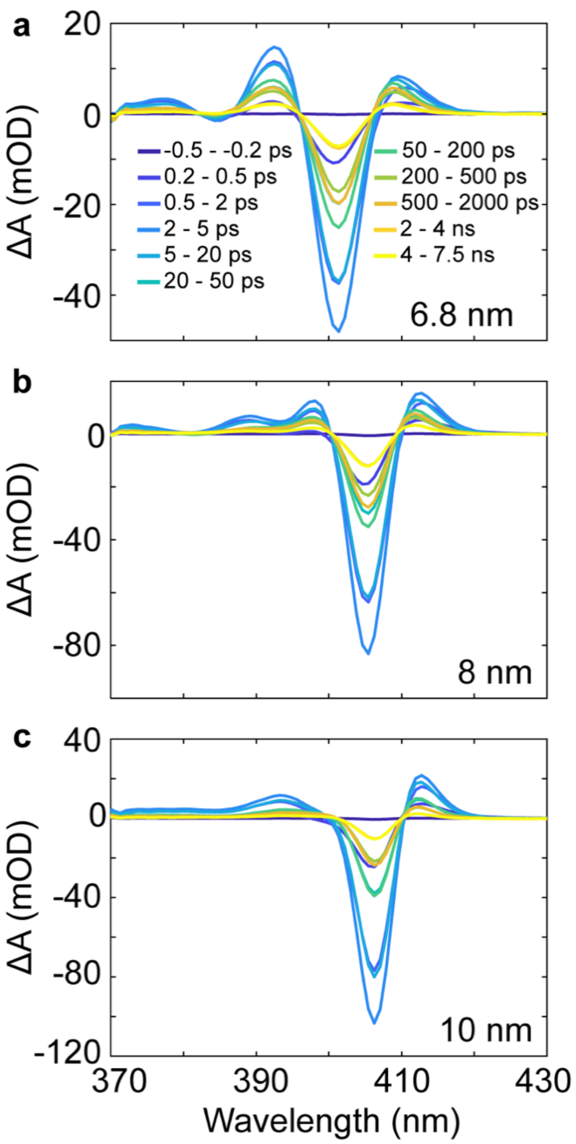

Fig. S9: Transient absorption spectra of (a) 6.8 nm edge length (b) 8 nm edge length and (c) 10 nm edge length $CsPbCl_3$ nanocrystals. In each case, the nanocrystals were excited at 343 nm with a pump fluence of 3 μJ/cm^2.

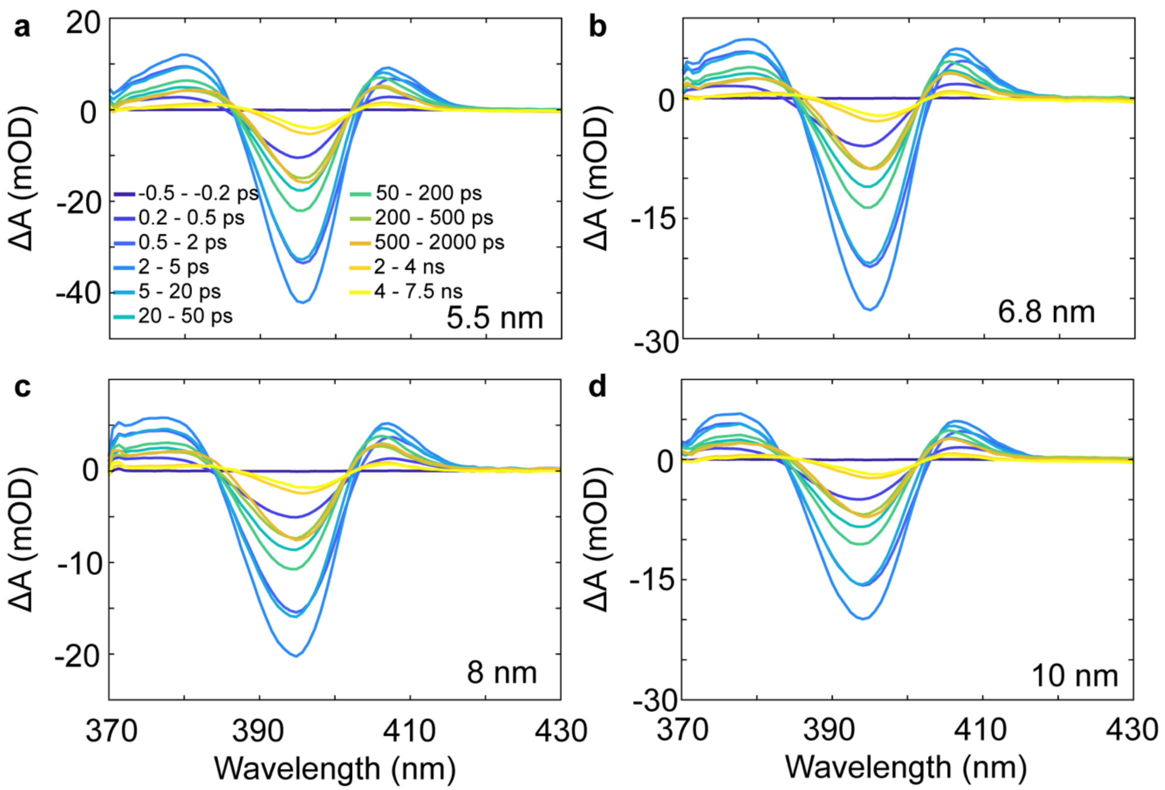

Fig. S10: Transient absorption spectra of (a) 5.5 nm edge length (b) 6.8 nm edge length (c) 8 nm edge length and (d) 10 nm edge length 1.8% Mn-doped $CsPbCl_3$ nanocrystals. In each case, the nanocrystals were excited at 343 nm with a pump fluence of 3 μJ/cm^2.

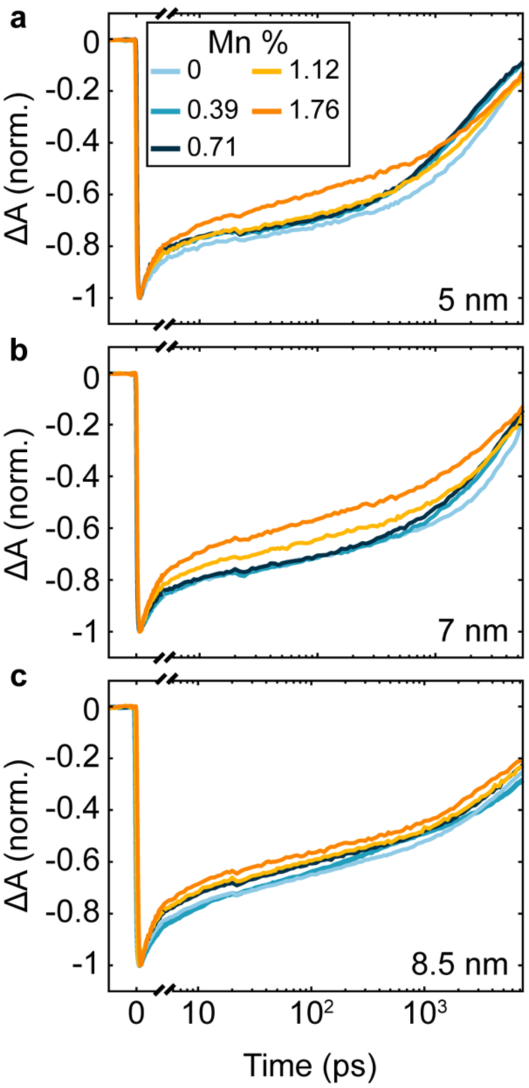

Fig. S11: Normalized transient absorption kinetic probed at the respective ground state bleach for $CsPbCl_3$ NCs with varying Mn dopant concentrations, with edge length sizes of (a) 5 nm, (b) 7 nm, and (c) 8.5 nm. The Mn dopant concentration for the largest size is given in the legend. All TA experiments were performed at a pump wavelength of 343 nm (ca. 100 fs pulses) with an excitation fluence of 3 μJ/cm^2.

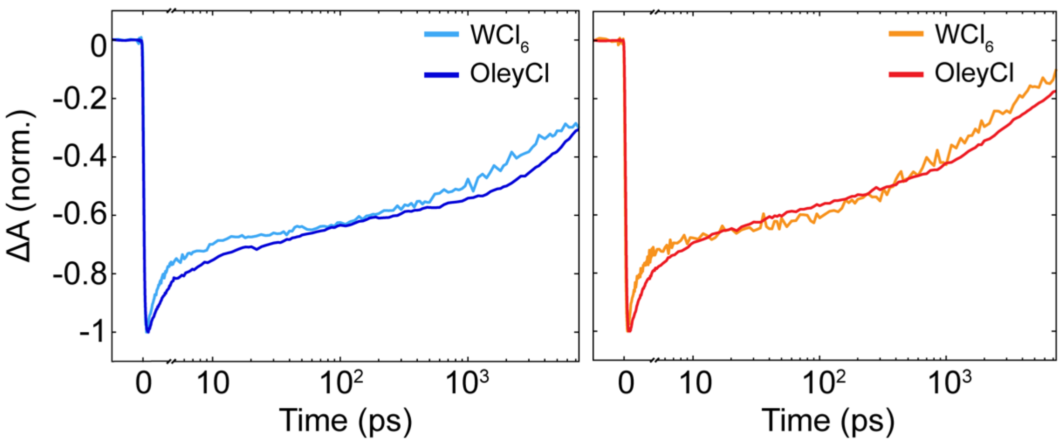

Fig. S12: Transient absorption kinetics extracted from the exciton ground-state bleach for undoped $CsPbCl_3$ nanocrystals (left panel) and 1.8% Mn-doped $CsPbCl_3$ nanocrystals (right panel), in each case chloride-shifted with either WCl_6 (light traces) or chloride-shifted with oleylammonium chloride (dark traces).

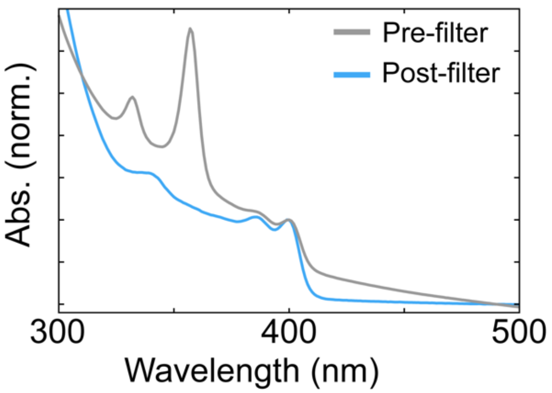

Fig. S13: Normalized absorption spectra of $CsPbCl_3$ nanocrystals chloride-shifted with oleylammonium chloride before (grey line) and after (blue line) syringe filtering, removing impurities.

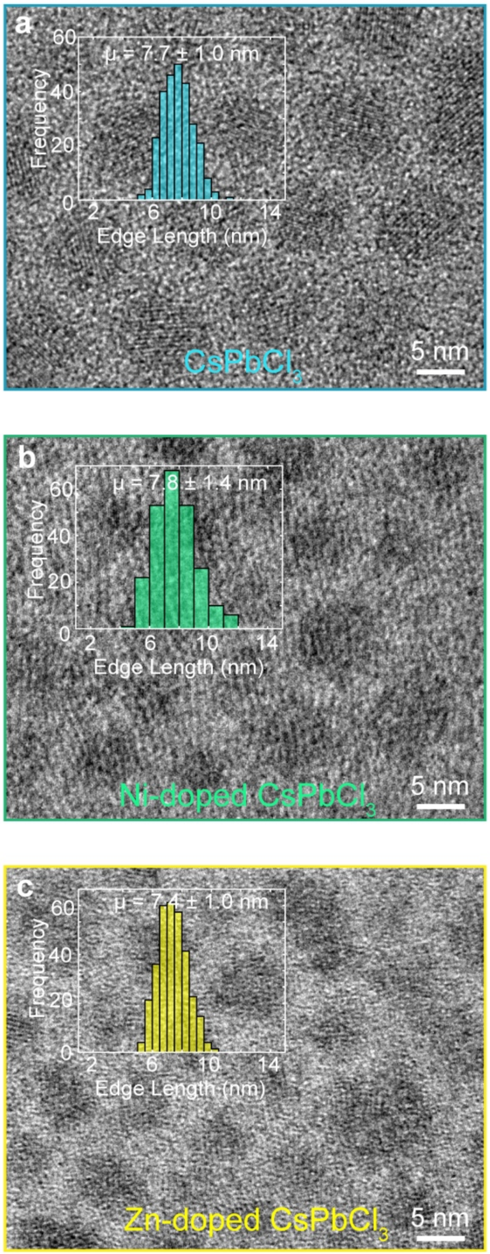

Fig. S14: TEM micrographs of (a) $CsPbCl_3$, (b) Ni-doped $CsPbCl_3$, and (c) Zn-doped $CsPbCl_3$ nanocrystals synthesized under otherwise identical conditions. In each case, the metal dopant to Pb molar feed ratio was 50:50. Nanocrystal sizes are histogrammed in the inset, incl. the mean nanocrystal size and standard deviation.

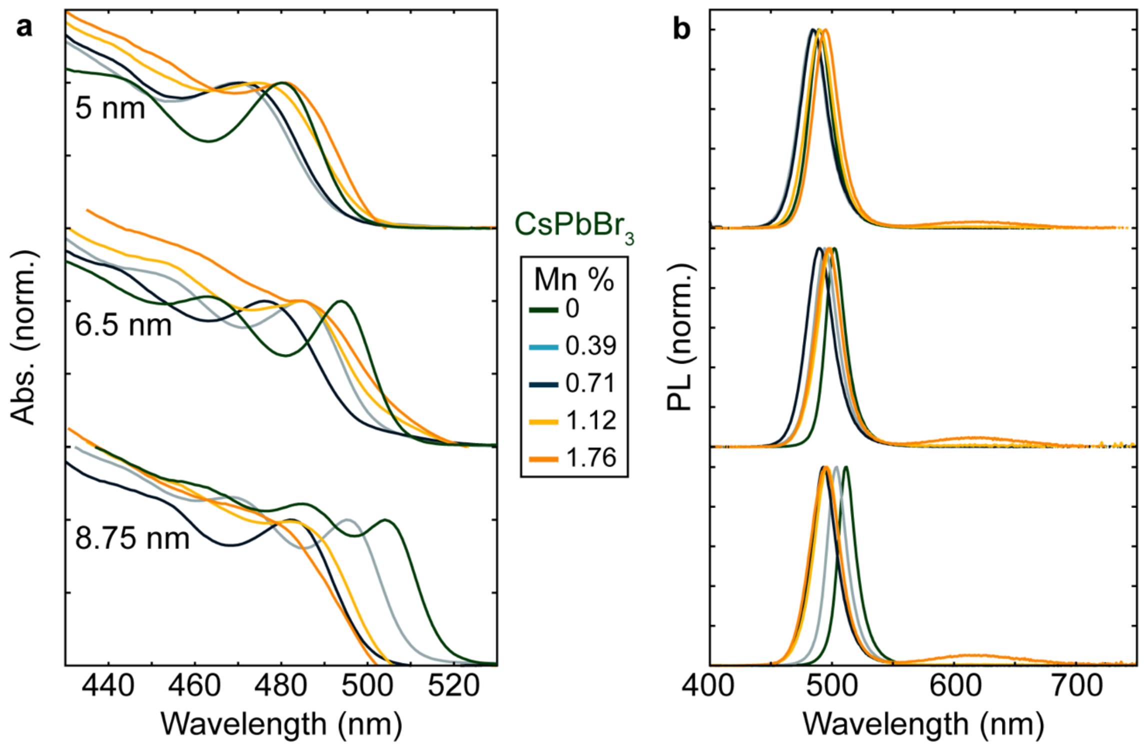

Fig. S15: Exemplary normalized (a) absorption and (b) PL emission spectra of three different sizes of $CsPbBr_3$ NCs with varying Mn dopant compositions. The Mn content of the largest NCs measured by ICP-MS is given in the legend.

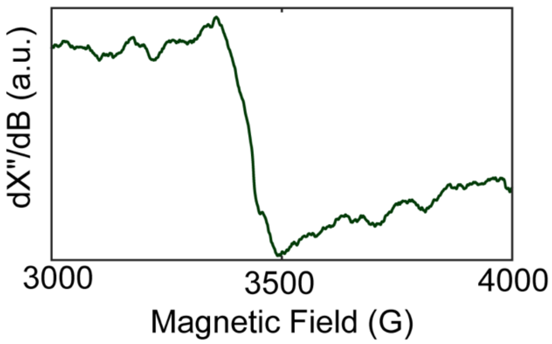

Fig. S16: X-band electron paramagnetic resonance data for Mn-doped $CsPbBr_3$ nanocrystals recorded at room temperature.

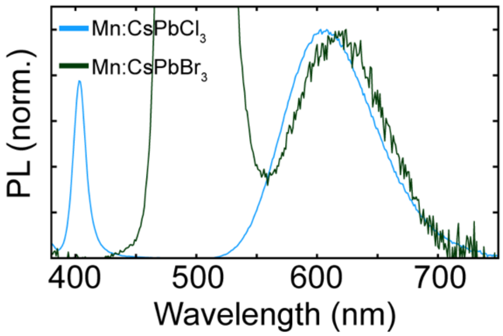

Fig. S17: Normalized PL spectra of Mn-doped $CsPbCl_3$ (blue) and Mn-doped $CsPbBr_3$ (green) NCs.

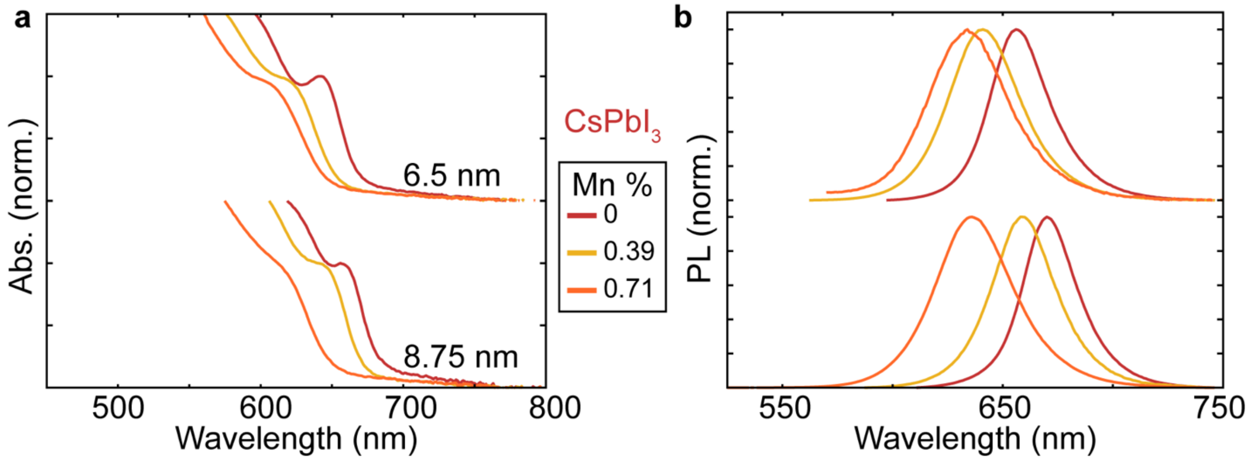

Fig. S18: Exemplary normalized (a) absorption and (b) PL emission spectra of two different sizes of $CsPbI_3$ NCs with varying Mn dopant compositions. The Mn content of the largest NCs measured by ICP-MS is given in the legend.

References

(1)Nedelcu, G.; Protesescu, L.; Yakunin, S.; Bodnarchuk, M. I.; Grotevent, M. J.; Kovalenko, M. V. Fast Anion-Exchange in Highly Luminescent Nanocrystals of Cesium Lead Halide Perovskites (CsPbX3, X = Cl, Br, I). *Nano Lett.* **2015**, *15* (8), 5635–5640. https://doi.org/10.1021/acs.nanolett.5b02404.

(2)Creutz, S. E.; Crites, E. N.; De Siena, M. C.; Gamelin, D. R. Anion Exchange in Cesium Lead Halide Perovskite Nanocrystals and Thin Films Using Trimethylsilyl Halide Reagents. *Chem. Mater.* **2018**, *30* (15), 4887–4891. https://doi.org/10.1021/acs.chemmater.8b02100.

(3)Luo, W.; Kim, S.; Lempesis, N.; Merten, L.; Kneschaurek, E.; Dankl, M.; Carnevali, V.; Agosta, L.; Slama, V.; VanOrman, Z.; Siczek, M.; Bury, W.; Gallant, B.; Kubicki, D. J.; Zalibera, M.; Piveteau, L.; Deconinck, M.; Guerrero-León, L. A.; Frei, A. T.; Gaina, P. A.; Carteau, E.; Zimmermann, P.; Hinderhofer, A.; Schreiber, F.; Moser, J.-E.; Vaynzof, Y.; Feldmann, S.; Seo, J.-Y.; Rothlisberger, U.; Milić, J. V. From Chalcogen Bonding to S–π Interactions in Hybrid Perovskite Photovoltaics. *Advanced Science* **2024**, 2405622. https://doi.org/10.1002/advs.202405622.

(4)Stoll, S.; Schweiger, A. EasySpin, a Comprehensive Software Package for Spectral Simulation and Analysis in EPR. *Journal of Magnetic Resonance* **2006**, *178* (1), 42–55. https://doi.org/10.1016/j.jmr.2005.08.013.

(5)Moreira, R. L.; Dias, A. Comment on “Prediction of Lattice Constant in Cubic Perovskites.” *Journal of Physics and Chemistry of Solids* **2007**, *68* (8), 1617–1622. https://doi.org/10.1016/j.jpcs.2007.03.050.
